# Inducible Nitric Oxide Synthase Polymorphisms and Nitric Oxide Levels in Individuals with Chronic Periodontitis

**DOI:** 10.3390/ijms18061128

**Published:** 2017-06-15

**Authors:** Raquel M. Scarel-Caminaga, Flávia F. Cera, Suzane C. Pigossi, Livia S. Finoti, Yeon J. Kim, Aline C. Viana, Rodrigo Secolin, Marcelo F. Montenegro, José E. Tanus-Santos, Silvana R. P. Orrico, Joni A. Cirelli

**Affiliations:** 1 Department of Morphology, School of Dentistry at Araraquara, São Paulo State University (UNESP), Humaita St., 1680, Araraquara CEP 14801-903, São Paulo, Brazil; flaviafcera@gmail.com; 2 Department of Diagnosis and Surgery, School of Dentistry at Araraquara, São Paulo State University (UNESP), Humaita St., 1680, Araraquara CEP 14801-903, São Paulo, Brazil; supigossi@ymail.com (S.C.P.); dentistaaline@yahoo.com.br (A.C.V.); s-orrico@foar.unesp.br (S.R.P.O.); cirrelli@foar.unesp.br (J.A.C.); 3 Department of Periodontics, School of Dental Medicine, University of Pennsylvania, 240 S 40th St., Philadelphia, PA 19104, USA; lifinoti@msn.com; 4 Department of Implantology, University of Santo Amaro, Professor Enéas de Siqueira Neto, St., 340, Santo Amaro CEP 04829-300, São Paulo, Brazil; yeon_jungkim@yahoo.com.br; 5 Department of Medical Genetics, University of Campinas—UNICAMP, Tessália Vieira de Camargo St., 126 Campinas CEP 13083-887, São Paulo, Brazil; rsecolin@gmail.com; 6 Department of Pharmacology, Ribeirão Preto Medical School, University of São Paulo, Bandeirantes Avenue, 3900, Ribeirão Preto CEP 14.049-900, São Paulo, Brazil; marcelofm@pq.cnpq.br (M.F.M.); tanus@fmrp.usp.br (J.E.T.-S.)

**Keywords:** genetic polymorphism, chronic periodontitis, nitric oxide, inducible nitric oxide synthase

## Abstract

This study aimed to investigate whether the −1026(A>C)(rs2779249) and +2087(A>G)(2297518) polymorphisms in the *NOS2* gene were associated with chronic periodontitis (CP) and with salivary levels of nitrite (NO_2_^−^) and/or nitrate + nitrite (NOx). A group of 113 mixed-race patients were subjected to periodontal, genetic, and biochemical evaluations (65 CP/48 periodontally healthy subjects). DNA was extracted from oral epithelial cells and used for genotyping by polymerase chain reaction (real-time). Salivary NOx concentrations were determined using an ozone-based chemiluminescence assay. Association of CP with alleles and genotypes of the −1026(A>C) polymorphism was found (*X*^2^ test, *p* = 0.0075; 0.0308), but this was not maintained after multiple logistic regression, performed to estimate the effect of covariates and polymorphisms in CP. This analysis demonstrated, after correction for multiple comparisons, that only the female gender was significantly associated with CP. Polymorphisms analyzed as haplotypes were not associated with CP. NOx levels were significantly higher in the control group of heterozygous individuals for both polymorphisms. In conclusion, the female gender was significantly associated with CP, and higher levels of salivary NOx were found in control subjects and associated with the heterozygous state of the *NOS2* polymorphisms, reinforcing the potential of NO metabolites as markers of periodontitis status.

## 1. Introduction

Periodontal diseases (PD), including chronic periodontitis (CP), the most common form of destructive PD in adults, are chronic inflammatory diseases demonstrating loss of connective tissue together with alveolar bone [1]. Bacterial infection is a primary cause of PD, but the disease progression in response to bacteria and metabolic products depends on the production of host inflammatory mediators [2,3]. Periodontitis is considered a complex disease, involving various biological pathways [4]. Complex diseases are associated with variations in multiple genes, each of which has a small overall contribution and relative risk for disease process [5]. Evidence for a genetic factor influencing the periodontal condition was obtained in a study enrolling twins [6]. The genetic makeup of an individual affected by PD together with the environmental influence determine the phenotype of the disease [4]. Most genetics research in periodontitis has focused on gene polymorphisms that play a role in immunoregulation, but little is known regarding a key short-lived molecule that plays role in many physiological and pathological processes [7]. This is nitric oxide (NO), which is a gaseous free radical generated from the conversion of l-arginine to l-citrulline by nitric oxide synthases (NOS) [8]. There are three isoforms of NO synthase (NOS): endothelial (eNOS), neuronal (nNOS), and inducible (iNOS) [9].

In regard to the pathogenesis of CP, evaluation of iNOS appears to be the most interesting, since it is expressed almost exclusively under inflammatory stimuli, such as bacterial lipopolysaccharides and proinflammatory cytokines, in a variety of cell types, including macrophages and neutrophils [10]. PD studies have reported increases in iNOS activity, indicating that in the disease process there might be the production and participation of NO [11]. NO mediates cytotoxic action against microbial pathogens [7] and cell-damaging and toxic effects, by forming peroxynitrite in a reaction with superoxide, which contributes to DNA damage as well as protein damage by combining with tyrosine to form nitrotyrosine (NT) [12,13,14].

An advance in our understanding of NO biology was the recognition of the nitrate–nitrite–NO axis, whereby nitrate (predominantly from dietary sources) could be converted to nitrite, and nitrite could be reduced to NO [15]. As a biomarker, salivary NO level can be utilized as a good indicator of the inflammatory status of the periodontium [16,17,18]. However, NO metabolites have a very short life, given that direct measurements of NO from tissues and biological fluids are difficult to perform, but nitrite and nitrate are the relatively stable end products of NO oxidation [19,20]. Interestingly, the level of NO biomarker and its end metabolites in saliva have been shown to be of more value in the assessment of periodontal health than their levels in the gingival crevicular fluid (GCF) [21]. However, salivary NO quantitation in patients with CP has demonstrated conflicting findings. Some studies have found higher levels of NO metabolites in patients with PD in comparison with healthy controls [7,11,22], while others have observed the opposite [23,24,25].

The production of NO by the activity of iNOS can be influenced by polymorphisms in the iNOS gene (*NOS2* gene, OMIM 163730) [26,27]. Both −1026(A>C) and +2087(A>G) single nucleotide polymorphisms (SNPs) in the *NOS2* gene are potentially functional [12,18,23,28,29] and clinically relevant, as they are associated with hypertensive disorders [29,30,31,32], migraine with aura [26], and malignant neoplasias [33,34].

When different SNPs are located in close physical proximity to each other on the chromosome and are inherited in blocks without recombination between parental chromosomes, they are called haplotypes [35]. The −1026(A>C)(rs2779249) and +2087(A>G)(2297518) SNPs in the *NOS2* gene were previously investigated as haplotypes in Brazilian [26] and Finnish populations [32]. Haplotypes are more powerful for detecting susceptibility alleles than individual polymorphisms; hence, in disease-association studies, they may give more conclusive results [35].

Because there is only one previous study investigating the association of the above-mentioned SNPs in the *NOS2* gene with CP [36], and considering that salivary NO levels in patients with CP remain controversial, we investigated in the present study the possibility of an association of susceptibility to CP with SNPs in the *NOS2* gene and its haplotypes and whether these patients’ genetic carriage could be related to salivary levels of nitrite (NO_2_^−^) and/or nitrate + nitrite (NOx).

## 2. Results

The power calculation we performed showed that a sample size of 113 individuals was large enough to detect a significant association between CP and the studied genetic polymorphisms, with an α-value of 0.025, OR = 2.65, and a power of 94%.

Table 1 summarizes demographic data on the mixed-race subjects, given our Brazilian multiethnic population. Comparing the two groups under investigation, there was similarity regarding mean age and gender (*p* > 0.05). There was a higher proportion of smokers in the CP group than in the control group, leading to a slight (*p* < 0.0465) but significant difference between the groups. Considering smoking habits by gender, although there was a higher percentage of female smokers in the CP group, the Fisher’s exact test demonstrated no association of these characteristics with CP (*p* = 0.6894).

Table 2 showed that allele and genotype frequencies of the −1026(A>C) SNP in the *NOS2* gene were significantly different between control and disease groups. For each studied group, the genotype distributions of each SNP were consistent with the assumption of Hardy–Weinberg equilibrium. The AC genotype of the -1026 SNP was more frequent in the CP group (41.5%, *p* = 0.0308) than in the control group. When we combined the AC and AA genotypes, we found that individuals carrying these genotypes were 2.44 times more susceptible to developing CP (*p* = 0.0341, OR = 2.44; 95% CI = 1.13–5.24, Table 2). The +2087(A>G) SNP in the *NOS2* gene demonstrated no significant findings. 

Table 3 shows the results of multivariate analysis, which was performed to more accurately evaluate the strength of any association and to eliminate the distortion caused by confounding effects. Multiple logistic regression analysis demonstrated that the −1026(A>C) SNP lost its significant association with CP after we adjusted for covariates (Table 3). The multiple logistic regression analysis demonstrated that gender and smoking habits were associated with CP (nominal *p* = 0.0004). However, after we adjusted for covariates, only gender maintained its significance as a confounding factor in the development of CP (corrected *p* = 0.0036, Table 3).

The haplotypes formed by the −1026(A>C) and +2087(A>G) SNPs showed a similar distribution between the control and CP groups. The most frequent haplotype in the controls was −1026(C) +2087(G) in homozygotes (CG/CG = 43.8%), followed by the two heterozygous haplotypes AG/CG = 22.9% and CA/CG = 16.7%.

Table 4 shows a significantly higher concentration of NOx (nitrate + nitrite) in the control group than in the CP group. Moreover, we observed that heterozygous individuals for each investigated SNP produced significantly higher concentrations of NOx in the control group when compared with the CP group.

## 3. Discussion

This is the first study, to our knowledge, that investigated the −1026(A>C) and +2087(A>G) SNPs in the *NOS2* gene, as well as their haplotypes, and NO production in patients with CP. The *NOS2* haplotypes were not associated with CP in this studied Brazilian population. Moreover, any significant association of the +2087 SNP with CP was verified in our southeastern Brazilian population. However, in a northeastern Brazilian population, the +2087(GG) genotype was recently associated with protection against the development of PD [36]. Contrasting results for a SNP association in case-control studies considering different populations are very common, mainly because of differences in the genetic carriage (ethnicity) or in the study design [37].

Regarding the −1026 SNP, we found that individuals carrying the (AC) or (AA) genotypes were more susceptible to CP. However, similar to other studies [36,38], this genetic association was not maintained after the adjustments for covariates in the multiple logistic regression analysis. Multiple logistic regressions were used here to evaluate the influence of common (and other potential) covariates, in the association of each *NOS2* SNP with CP. These multiple comparisons revealed that only the female gender (after apply the Bonferroni’s correction) was significantly associated with CP. Thus, in this population, a factor (gender) other than genetic background was more powerful in predisposition to CP. Gender is a well-known risk factor for periodontitis, and the literature presents epidemiologic studies showing higher prevalence and severity of destructive PD in men than in women, even after adjusting for behavioral and environmental factors [39,40,41]. The effect of gender on PD can be attributed to the differences in gene expression and immune cell functioning modulated by sex steroid hormones or to physiological differences between the genders [42].

The levels of NO metabolites were verified to vary depending on the gender, usually being higher in males than in females [43,44]. Andrukhov et al., 2013 [45] observed that male patients with periodontitis exhibited significantly lower salivary NO_2_ and serum NOx levels. This data suggests that gender might influence the NO production in periodontitis. Interestingly, Skaleric et al., 2006 [46] and Han et al., 2013 [18] observed a positive link between NO and periodontitis, which was more pronounced in females. 

An increasing number of studies have investigated levels of nitrite and nitrate in saliva as periodontal disease biomarkers [16,17,18,23]. Higher nitrite concentrations were found in whole saliva of periodontally healthy subjects in comparison with PD patients, and this situation remained unchanged after periodontal treatment [23]. In the present study, we demonstrated higher salivary NOx concentrations in the control group than in the periodontitis group, which is in agreement with other studies [23,24,25,45]. Conversely, some studies have observed the opposite; i.e., lower salivary NO levels in the healthy group in comparison with the periodontitis group [7,11,18,22]. These contradictory results might be explained by the diverse methods used to quantify NO, their sensitivities [23], the unknown NO stability in saliva, an insufficient number of patients, and different clinical parameters used to classify patients affected by PD [37]. 

Interestingly, we also observed a significantly higher number of healthy individuals who were heterozygous for the −1026(A>C)(rs2779249) and +2087(A>G)(rs2297518) SNPs producing higher levels of NOx (Table 4). We speculate that the observed higher salivary NOx concentrations produced by heterozygous individuals in the control group (Table 4) reflects a level of NO production that is sufficient to eliminate periodontal infection by periodontopathogens, given the antimicrobial activity of NO [7]. Ozer et al., 2011 [24] speculated that a decrease in salivary NO levels in patients with periodontitis compared with healthy individuals may lead to a reduction in the antibacterial properties of saliva, increasing the susceptibility of periodontal tissues to periodontopathogens [24,47]. The same authors [24] highlighted that the saliva of patients with periodontitis did not stimulate, but rather depressed NO synthesis in polymorphonuclear leukocytes (PMNL), whereas the saliva of healthy individuals stimulated NO synthesis [48]. Conversely, it is known that excessively high amounts of locally produced NO exceed the cytotoxic action against microbial pathogens and elicit proinflammatory molecules, unbalancing the immune response equilibrium and leading to periodontal tissue destruction [7]. Ozer et al., 2011 [24] mentioned that, when locally produced in high concentrations, NO may act as both a cytotoxic molecule against invading pathogens and against adjacent tissue cells, thus being related to both beneficial and harmful effects [49], and we entirely agree with them. Finally, the same authors [24] concluded that lower and higher concentrations of NO can initiate destruction in periodontal tissues, yet a threshold NO level might be required for the remodeling of periodontal tissues.

Indeed, although the investigated SNPs in the *NOS2* gene were demonstrated as functional [23,27,45], the exact molecular mechanisms of their action in the modulation of NO metabolites remain unknown. As suggested by Wang et al., 2013 [28] besides the SNPs investigated here, we cannot exclude the possibility of the presence of other functional polymorphisms in linkage disequilibrium (LD) with an investigated SNP being responsible for the genotype-dependent differences in iNOS activity. Further studies are needed to identify polymorphisms in this region and clarify which polymorphism(s) may possess the functional consequence(s) for the genotype-dependent differences in iNOS activity (e.g., NO levels), and provide mechanistic plausibility for the observed association between *NOS2* and susceptibility to CP. Moreover, other genes—such as the endothelial nitric synthase (eNOS), which also have polymorphisms—could potentially influence NO levels (even though NO production by eNOS is significantly lower than by iNOS). Indeed, other genes, mechanisms, and pathways could influence salivary NOx production and could be involved in CP development. Therefore, more studies are necessary to elucidate the role of different genes in the synthesis of NO (and its concentration in saliva) in the context of CP. 

In addition, other factors could also be influencing the salivary NO metabolites levels: physiological variables, hormones, salivary flow rate, and environmental exposures, such as diet since nitrite and nitrate are present in preserved foods and in green leafy vegetables [9]. Ingested nitrate is absorbed in the stomach and intestines and secreted in concentrated levels in the salivary glands [50]. This concentrated nitrate is reduced to nitrite via facultative anaerobic bacteria in the oral cavity [51]. Unfortunately, knowledge is lacking regarding the exact mechanisms of NO synthesis and action in the periodontium and saliva. 

Unfortunately, as a limitation of our study, we did not measure the salivary flow rate of the participants. Although it could have a potential effect on the detected salivary NOx levels, we hypothesize that the NOx concentrations found here were related to the immunological function that contributes to periodontal health. Regarding to the number of patients investigated here, 113 patients demonstrated sufficient power. A previous genetic study of haplotypes formed by the SNPs rs2779249 and rs2297518 [26] demonstrated a high OR value (OR = 2.65, 95% CI = 1.34–5.22). Therefore, we could assume that these SNPs have a large effect, and evidence of a genetic association could be detected with a smaller number of individuals. Nevertheless, further studies with larger sample and ethnically different populations, as well as meta-analysis approaches, are needed to better evaluate the potential association of polymorphisms in the *NOS2* gene and its influence on NO production in the development of periodontal disease.

## 4. Methodology

### 4.1. Selection of Subjects

This study involved individuals from the State of São Paulo in the southeastern region of Brazil. A total of 113 unrelated subjects, all with similar socio-economic status, were recruited from the patient pool of the School of Dentistry at Araraquara, São Paulo State University (UNESP), from November 2004 to May 2007. The study was approved by the Committee for Ethical Affairs of the São Paulo State University (Protocol number 57/04). All volunteers were informed of the aims and methods of this study, and all gave their written consent [35].

All subjects had to be in good general health, and the exclusion criteria were as follows: use of prophylactic antibiotics, chronic usage of anti-inflammatory drugs, current pregnancy, ongoing orthodontic therapy, and self-declared history of diseases that influence the immune system, diabetes mellitus, HIV infection, or immunosuppressive chemotherapy [35]. Information on smoking status was obtained using a self-reporting questionnaire. Smoking status was assessed, and subjects were classified as “smokers” or “nonsmokers”, according to Kornman et al., 1997 [48]. Smokers were current smokers, and nonsmokers were subjects who had never smoked or who had not smoked within the last five years. Each subject was examined by one of two calibrated periodontists (YJK and ACV) who carried out the periodontal examinations throughout the study period (weighted kappa = 0.74, considering the probing depth [PD]). The following clinical signs and parameters were assessed at six sites around each tooth: PD (as the distance from the gingival margin to the base of the periodontal pocket) and clinical attachment level (CAL, as the distance from the cement-enamel junction to the base of the periodontal pocket) measured to the nearest millimeter by a periodontal probe with Williams markings (Trinity-Campo Mourão, Brazil), and bleeding on probing (BOP), registered as present or absent, as a percentage of the total number of sites. These measurements were made in millimeters and were rounded down to the nearest whole millimeter. All fully erupted teeth, except third molars and retained roots, were examined. The periodontal diagnosis of subjects was established on the basis of clinical criteria, which categorized subjects into two groups, similar to previously published studies [35,52,53,54]:

- Control group (*n* = 48): subjects exhibiting no sites with CAL and PPD ≥ 3 mm and BOP.
- Periodontitis group (*n* = 65): subjects exhibiting one or more sites with CAL and PPD ≥ 3 mm and BOP.

Statistical power was calculated according to the strength of the single nucleotide polymorphism (SNP)-associated genetic disease in terms of the odds ratio (OR), the statistical test to be used, and the number of polymorphisms to be analyzed in the gene [55]. To verify the statistical power of our sample to detect genetic associations, we used the G*Power 3 software [56] with the following parameters: logistic regression; post hoc; two-tail; odds ratio (OR) = 2.65 [26] α error probability = 0.025 (because we investigated 2 SNPs) [26].

### 4.2. Analysis of Genetic Polymorphisms

Buccal epithelial cells from the subjects were obtained using 3 mL of 3% glucose mouthwash for 2 min. DNA was extracted with sequential phenol/chloroform/isoamyl alcohol (25:24:1) solution and precipitated with salt ethanol solution [35]. The analysis of genetic polymorphisms was performed using real-time polymerase chain reaction (qPCR). The −1026(A>C) (rs2779249) and +2087(A>G) (rs2297518) SNPs in the *NOS2* gene were genotyped using predesigned TaqMan SNP Genotyping Assays (Applied Biosystems, Foster City, CA, USA). DNA amplification was carried out in a 13 µL volume containing 20 ng genomic DNA, 6.25 µL of 2× Master Mix (Applied Biosystems), 0.63 µL 20× TaqMan Assay Mix (Applied Biosystems), and 4.63 µL of RNA/DNAase-free water (Applied Biosystems). The amplifications in 96-well plates were made in a 7500 Real Time PCR System (Applied Biosystems, Foster City, CA, USA). Subsequently, end-point fluorescence was determined using 7500 System Sequence Detection Software (Applied Biosystems, Foster City, CA, USA).

### 4.3. Measurement of Salivary NOx Concentration

The participants were asked not to rinse their mouth, brush their teeth, eat food, or drink liquids for 2 h before the time of clinic attendance (before the periodontal examination). Approximately 1 mL of non-stimulated saliva was collected from each patient and maintained on ice, until storage at −80 °C. 

Saliva aliquots were analyzed in triplicate for their nitrite (NO_2_^−^) and/or nitrate + nitrite (NOx) contents using an ozone-based chemiluminescence assay (NO analyzer, Sievers Model 280, Boulder, CO, USA). Details of the method were previously described [57,58].

### 4.4. Data Analysis

We used the chi-squared test to verify differences between cases and controls for gender and smoking habits, whereas the mean age difference between groups was evaluated using a Student’s *t*-test. A Fisher’s exact test was used to compare smoking habits by gender between control and CP groups. The distribution of each polymorphism in each group was tested for Hardy–Weinberg equilibrium. The haplotype analysis was made by the ARLEQUIN program^32^. Differences between groups in the allelic and genotypic frequencies of the polymorphisms and haplotypes were analyzed by the *X*^2^ test and odds ratio using the GraphPad InStat software, version 3.05 (Graph Pad Software Inc., San Diego, CA, USA). A multivariate logistic regression model was performed to estimate the effect of the covariates (age, nitrite, NOx, gender, smoking habits, and polymorphisms) and the SNPs in the phenotype, by R statistical software (https://www.r-project.org/). For the NOx concentration, the Shapiro–Wilk test for normality evaluation was performed, followed by the Kruskal–Wallis or Mann–Whitney tests for comparison between Control and CP groups. All tests were performed at a significance level of 5%, and the Bonferroni correction for multiple tests was applied.

## 5. Conclusions

After multiple logistic regression analysis, we concluded that neither the −1026(A>C) and +2087(A>G) SNPs in the *NOS2* gene, nor the haplotypes, but only gender were associated with CP. Moreover, there was association of the heterozygous −1026(AC) and +2087(AG) SNPs with a significantly higher concentration of NOx in Brazilian individuals not affected by CP. These results contribute to the investigation of the usefulness of salivary NO metabolites as markers of periodontitis status. More ambitiously, in the future, these markers can be associated with the patient’s genetic carriage, identifying individuals more susceptible to CP who can be benefit from preventive treatments. 

## Figures and Tables

**Table 1:** Demographic characteristics of the study participants.

Characteristics	Control	Chronic Periodontitis	Total	p *
	(*n* = 48)	(*n* = 65)	(*n* = 113)	
Age (Mean ± SD)	35.74 (±8.08)	41.84 (±8.43)	39.18 (±8.75)	0.1268
Gender (%)				
Female	23 (47.92%)	38 (58.46%)	61 (53.98%)	0.3572
Male	25 (52.08%)	27 (41.54%)	52 (46.02%)	
Smoking habits (%)				
Smokers	8 (16.66%)	23 (35.40%)	31(27.43%)	0.0465
Nonsmokers	40 (83.33%)	42 (64.60%)	82 (72.57%)	
Smoking habits by gender (%)				
Smokers	Female 4 (50.0%)	Female 14 (60.9%)	Female 18 (58.0%)	0.6894
	Male 4 (50.0%)	Male 9 (39.1%)	Male 13 (41.9%)	
Nonsmokers	Female 19 (47.5%)	Female 24 (57.1%)	Female 43 (52.4%)	0.5073
	Male 21 (52.5%)	Male 18 (42.9%)	Male 39 (47.6%)	

*p* * = comparison between control and chronic periodontitis groups. SD = standard deviation.

**Table 2:** Allele and genotype frequencies of the investigated SNPs in the *NOS2A* gene.

SNP	Control (%)	Chronic Periodontitis (%)	*p*
**−1026 (A>C)rs2779249**			
Allele	*n* = 96	*n* = 130	
A	22 (22.92%)	53 (40.77%)	0.0075
C	74 (77.08%)	77 (59.23%)	
	OR = 2.3	CI (95%) = 1.28–4.17	
Genotype	*n* = 48	*n* = 65	
AA	3 (6.3%)	13 (20.0%)	
AC	16 (33.3%)	27 (41.5%)	0.0308
CC	29 (60.4%)	25 (38.5%)	
*H-W Equilibrium*	*p* = 0.6954	*p* = 0.2593	
AA + AC	19 (39.58%)	40 (61.54%)	0.0341
CC	29 (60.420%)	25 (38.46%)	
	OR = 2.44	CI (95%) = 1.13–5.24	
**+2087 (A>G)rs2297518**			
Allele	*n* = 96	*n* = 130	
A	15 (15.6%)	20 (15.4%)	0.8913
G	81 (84.4%)	110 (84.6%)	
Genotype	*n* = 48	*n* = 65	
AA	1 (2.1%)	2 (3.1%)	
AG	13 (27.1%)	16 (24.6%)	0.9155
GG	34 (70.8%)	47 (72.3%)	
*H*-*W Equilibrium*	*p* = 0.8507	*p* = 0.6601	

SNP = single nucleotide polymorphism; OR = odds ratio; CI = confidence interval.

**Table 3:** Logistic regression results of the analysis.

Covariate		OR (95% CI)	*p*	
			Nominal	Corrected
Age		1.08 (1.0–1.18)	0.0485	0.4365
Nitrite concentration		1.00 (0.98–1.02)	0.9873	1.0000
NOx concentration		0.99 (0.97–1.01)	0.1825	1.0000
Gender	Male	Reference category	-	
	Female	8.35 (2.23–31.27)	0.0004	0.0036 *
Smoking Habits	Nonsmokers	Reference category	-	
	Smokers	4.35 (1.1–17.22)	0.0242	0.2178
SNP −1026	CC	Reference category	-	
	AC	2.99 (0.77–11.53)	0.1023	0.9207
	AA	5.03 (0.92–27.57)	0.0428	0.3852
	C	Reference category	-	
	A	2.95 (0.91–9.58)	0.0653	0.5877
SNP +2087	AA	Reference category	-	
	AG	2.84 (0.09–89.14)	0.4797	1.0000
	GG	7.01 (0.21–228.71)	0.1860	1.0000
	A	Reference category	-	
	G	2.70 (0.66–11.01)	0.1527	1.0000

OR = odds ratio; CI = confidence interval. * *p*-value which maintained statistically significant after Bonferroni’s correction.

**Table 4:** Salivary NOx and Nitrite concentrations comparisons between Control and CP groups

Mensuration	NOx (µM)			Nitrite (nM)		
	Control	CP	*p*-Value *	Control	CP	*p*-Value
	(*n* = 48)	(*n* = 65)		(*n* = 48)	(*n* = 65)	
Mean	52.19	26.52		36.78	30.41	
Standard Deviation	±57.27	±33.76		±39.13	±35.68	
Median (Min–Max)	27 (0–200)	12 (0–160)	**0.009**	19 (2–146)	18 (0–150)	0.368
−1026 (A>C)rs2779249	Median (Min–Max)			Median (Min–Max)		
AA	6 (5–14)	7 (0–83)	0.78	30 (19–135)	22 (0–135)	0.42
AC	35 (5–193)	14 (0–160)	0.01	24 (4–122)	18 (0–150)	0.13
CC	27 (0–200)	16 (1–75)	0.37	17 (2–146)	19 (1–144)	0.98
*p*-value ^#^	0.11	0.67		0.23	0.69	
+2087 (A>G)rs2297518	Median (Min–Max)			Median (Min–Max)		
AA	22	28.5 (0–57)	1.00	14	35.5 (9–62)	1.00
AG	38 (6–200)	6.5 (0–55)	0.004	30 (3–146)	15.5 (1–135)	0.13
GG	27 (0–170)	16 (0–160)	0.12	19 (2–131)	20 (0–150)	0.88
*p*-value ^#^	0.31	0.07		0.45	0.49	

NOx = nitrate + nitrite, Shapiro–Wilk test for normality evaluation (showing non-parametric data). Min = Minimum, Max = Maximum), *p*-value * = comparison between Control and CP (Mann–Whitney test), *p*-value ^#^ = comparison among genotypes into the Control or CP groups (Kruskal–Wallis test).
